# Effect of smoking on dental pulp responses to thermal and electrical sensibility tests

**DOI:** 10.18332/tid/224444

**Published:** 2026-07-16

**Authors:** Emre Çulha, Özge Yüksekkaya, İrfan Yüksekkaya, Oğuz Burhan Çetinkaya

**Affiliations:** 1 Department of Endodontics, Dentistry Faculty, Gaziantep University, Gaziantep, Türkiye; 2 Şehitkamil Oral and Dental Health Center, Gaziantep, Türkiye

**Keywords:** smoking, electric pulp tester, thermal pulp test, pulp vitality, tobacco

## Abstract

**INTRODUCTION:**

Smoking is associated with vascular constriction and neural alterations that may influence pulpal blood flow and sensory responses. However, the relationship between cigarette consumption and dental pulp sensibility to thermal and electrical stimuli remains incompletely understood. This cross-sectional study aimed to evaluate the association between daily cigarette consumption and the dental pulp sensibility of maxillary central incisors assessed using a cold test and electric pulp test (EPT).

**METHODS:**

Eighty-eight systemically healthy participants aged 18–40 years were allocated into four groups according to daily cigarette consumption: non-smokers, 1–10, 11–20, and >20 cigarettes/day (n=22 per group). Pulp sensibility was assessed using EPT (stimulus range: 0–64) and cold testing (response time in seconds). Group comparisons were performed using Kruskal–Wallis and Mann–Whitney U tests, followed by Dunn’s post hoc analysis where appropriate (p<0.05).

**RESULTS:**

Mean EPT stimulus thresholds increased progressively with higher daily cigarette consumption, ranging from 4.27 ± 1.32 in non-smokers to 7.23 ± 1.80 in heavy smokers (p=0.001). Similarly, cold test response times were significantly prolonged with increasing cigarette intake (0.51 ± 0.31 s in non-smokers vs 1.07 ± 0.33 s in heavy smokers; p=0.001). In contrast, smoking duration was not significantly associated with either EPT thresholds (p=0.832) or cold test response times (p=0.258).

**CONCLUSIONS:**

Dental pulp sensibility responses were increasingly attenuated with higher daily cigarette consumption, whereas smoking duration showed no significant association with test outcomes. These findings suggest that the daily quantity of cigarettes consumed may be a more relevant factor than smoking duration when interpreting pulp sensibility test results. Clinicians should consider smoking status as a potential modifier during pulp sensibility assessment.

## INTRODUCTION

Pulp vitality status is crucial in endodontics since it is an essential component of diagnostic treatment planning^1^. The most reliable method for assessing pulp vitality is to evaluate it via histological sections. However, because the pulp is surrounded by a calcified obstacle, this needs to be performed before initiating endodontic treatment^2^. Unlike histological examination, which is the absolute gold standard but impractical *in vivo* due to the enclosed nature of the pulp chamber, thermal and electrical tests allow clinicians to infer pulpal nerve responsiveness indirectly. These tests remain the most widely used diagnostic tools in clinical endodontics due to their non-invasiveness, ease of use, reproducibility, cost-effectiveness, and rapid feedback capabilities. Thermal tests, including hot and cold methods, activate the pulpal nerves via the flow of dentinal fluid at varying temperatures, which leads to the movement of the odontoblast segments and, therefore, physically activates the pulpal nerves^3^. Dry ice, ice, and coolant sprays, including tetrafluoromethane, butane, propane, isobutane, dichlorofluoromethane, and ethyl chloride, are among the materials that are available for cold tests^4^. The electric pulp test (EPT) is a well-established diagnostic modality used to assess pulpal sensibility by applying a gradually increasing electric stimulus to the tooth surface. The primary strategy of the EPT involves using electrical stimuli to cause an ionic shift across the neural membrane, which affects the action potential by causing a quick jump at the nodes of Ranvier in myelinated neurones^5^. Clinically, EPT offers several advantages: it provides quantitative threshold values, enabling repeatable and longitudinal comparisons; it is noninvasive, rapid, costeffective, and generally well-tolerated by patients^3^.

Smoking is a global public health problem leading to many systemic diseases^6^. The promoting characteristics of nicotine are the major factor perpetuating smoking behavior, which is a complex feature impacted by both genetic and environmental factors^7^. The duration and severity (the amount of tobacco products consumed) of the dependence determine the negative effects of smoking. Nicotine, one of the numerous toxic chemicals in tobacco, has a negative impact on the immunological, gastrointestinal, cardiovascular, respiratory, and reproductive organs^8^. Smoking impacts the vascular system by lowering oxygen and nutritional status, reducing pulp defenses, and causing necrosis^9^. The harmful consequences of smoking are well understood, but research specifically addressing its impact on pulpal sensibility remains limited. Nicotine and other tobacco constituents induce vasoconstriction and reduced pulpal blood flow, thereby altering osmotic pressure and fluid dynamics within the tissue – a mechanism that may interfere with neural activation during sensibility testing^10^. Additionally, smoking has been linked to changes in neuropeptide expression and local immune modulation within the pulp, including increased levels of calcitonin gene-related peptide and decreased antimicrobial peptides, which may impair nociceptive sensation^11^. These biological alterations suggest that smokers could exhibit prolonged or blunted responses to thermal and electrical sensibility tests, yet few clinical studies have systematically evaluated this possibility.

Consequently, a clear gap exists in the literature: while the systemic implications of smoking on healing and vascular health are acknowledged, its specific effects on pulpal neural responsiveness and sensibility testing remain underexplored. Hence, our current investigation is uniquely positioned to fill this gap by comparing EPT and cold test responses between smokers and non-smokers, thereby providing a controlled evaluation of how varying levels of cigarette consumption may influence pulpal sensibility.

The aim of our study was to evaluate the effect of cigarette consumption on the pulpal response of maxillary central incisors subjected to cold testing and EPT. The null hypothesis of the study was that smoking would be unable to change pulp sensibility test responses.

## METHODS

### Study design and participants

Within this cross-sectional study, the sample size was determined using the statistical program G*Power version 3.1 (Universität Kiel, Kiel, Germany). The primary outcome used for the calculation was the between-group difference in EPT stimulus thresholds, which exhibits lower intra-individual variability compared with cold test response times and is commonly used as a reference outcome in pulp sensibility research. A moderate effect size (f=0.35) (Cohen’s d) was set at 0.65, based on a previous study that evaluated pulp sensibility responses in anemic and healthy women^12^. With an alpha level of 0.05 and a desired statistical power of 0.80, the minimum sample size required per group was calculated to be 20 participants (20 non-smokers and 60 smokers). Based on these parameters, a minimum total sample size of 84 participants was required. To account for potential exclusions or incomplete data, 88 participants (22 subjects per group) were ultimately included in the study to ensure robust statistical analysis. The chosen effect size reflects a moderate to large expected difference in pulp response values between smokers and non-smokers. This approach ensures that the study is adequately powered to detect meaningful differences between groups; therefore, smaller differences between groups may not have been identified.

### Inclusion criteria

Participants aged 18–40 years were included in the study. Individuals aged >40 years were excluded to minimize the potential influence of age-related changes in pulpal structure and neural responsiveness, as aging is associated with reduced pulp volume, vascularity, and sensibility^13^. Only systemically healthy individuals with good oral hygiene, defined as a plaque index below 20%, were enrolled. Eligible teeth were required to exhibit favorable periodontal conditions, characterized by a clinical attachment loss of ≤5 mm and marginal bone loss of less than one-third of the root length. All participants were required to be capable of maintaining oral hygiene and to provide written informed consent prior to participation.

### Exclusion criteria

Individuals aged >40 years, pregnant participants, and those with current or recent (within the previous five days) use of analgesics or pain-modifying medications were excluded. Participants who were immunocompromised or receiving long-term corticosteroid therapy were also not eligible for inclusion. Teeth presenting with internal or external resorption, radiographically undetectable pulp space, a history of dental trauma, recent orthodontic treatment, restorations, crowns, or previous root canal therapy were excluded from the analysis. In addition, passive smokers and former smokers were excluded to avoid misclassification bias related to tobacco exposure. Participants with any chronic systemic disease or immune compromise were also excluded from the study.

### Smoking classification

Each participant was assigned a unique identification number, which was changed to data after the study to prevent bias. As a result, the assessors were unable to determine whether the participants smoked or not. Smoking status was classified according to internationally accepted definitions provided by the World Health Organization and the Centers for Disease Control and Prevention^14^. The patients were divided into four equal groups (n=22) based on how many cigarettes they smoked each day: non-smokers, 1–10, 11–20, and >20. This categorization has been widely used in previous clinical and epidemiological studies to evaluate dose-dependent effects of smoking^15,16^. Participants who had never smoked more than one cigarette per 1 to 3 days were classified as non-smokers. Participants were considered smokers if they answered yes to at least one of these questions: ‘Have you smoked at least 100 cigarettes in your lifetime?’ and ‘Do you currently smoke?’. The locations of the tested teeth and information on smoking behaviors were obtained.

### Variables and pulp testing

The sensibility tests were performed on the upper central incisors. The teeth’s exterior was cleared of debris, calculus, and plaque before testing. After using a cotton roll to dry and isolate the teeth, a gel (BP Ultra Gel, Turkuaz, Türkiye) was applied as a contact medium to the enamel. An EPT device probe (Digitest, Parkell, Edgewood, NY, USA) was placed on the coronal third of the labial surface of the teeth, and the lip clip was placed in the participant’s mouth. The participant was then instructed to notify the assessor of any tingling, discomfort, or other sensations experienced while the EPT device was being activated. The number between 0 and 64 indicating the current of the EPT device was recorded as the participant’s pulpal response. Device data obtained from the EPT were classified as 0–6 and 7–13, and response times to the cold test were classified as <1 s and 1–2 s. These categories were created for descriptive purposes only and did not represent clinical thresholds. To reduce the impact of one test on the other, the sensibility tests were conducted one to two minutes apart. Cold testing with butane, propane, and isobutane (Galena, Gordona, Italy) was performed using a cotton bud on the labial surface of the crown until the participant gave a reaction to the same calibrated examiner. A digital stopwatch was used to monitor and record the participant’s response time to the cold stimuli.

### Statistical analysis

Descriptive statistics of the data collected included mean and standard deviation, and median and interquartile range (IQR) for numerical parameters, as well as frequencies and percentages for categorical variables. All statistical tests were two-tailed, and the level of significance was set at p<0.05. The Shapiro-Wilk test was used to determine if numerical variables conformed to a normal distribution. These variables were compared based on the study groups using the Kruskal-Wallis test for categorical variables with three or more groups and the Mann-Whitney U test for those with two groups. To ascertain which group was responsible for the difference between the groups, Dunn’s multiple comparison test was employed. Additionally, chi-squared analysis was used to assess the differences between categorical variables. The SPSS 22.0 software was used to conduct the analyses.

## RESULTS

A total of 88 participants were included in the analysis, of whom 27 (31%) were female and 61 (69%) were male. The mean age of the study population was 25.4 ± 4.2 years (range: 18–35) (Table 1). Among smokers (n=66), the mean duration of smoking habit was 5.1 ± 3.6 years, with 53% (n=35) reporting a smoking duration of 0–5 years and 47% (n=31) reporting 6–10 years (Table 2). There was no statistically significant difference in gender distribution among the smoking groups (p=0.07). Participants were evenly distributed across smoking categories, with 22 individuals (25%) in each group. No statistically significant differences were observed with the independent samples t-test in EPT thresholds or cold test response times between male and female participants (p>0.05).

**Table 1:** Comparison of duration of smoking habit with sensibility test results, mean age and smoking habit duration

*Variables*	*Smoking habit duration (years)*	*Mean ± SD*	*Median (IQR)*	p*
**Electric pulp test** (device scale units)	0–5	6.6 ± 1.77	7 (5–7)	0.832
	6–10	6.52 ± 1.06	7 (6–7)	
**Cold test response time** (seconds)	0–5	0.9 ± 0.33	0.83 (0.68–1.05)	0.258
	6–10	0.94 ± 0.26	0.98 (0.71–1.15)	
**Age** (years) (N=88)		25.42 ± 4.2	24 (18–35)	
**Smoking habit duration** (N=66)		5.09 ± 3.55	5 (1–15)	

* Mann-Whitney U test. IQR: interquartile range.

**Table 2:** Comparison of sensibility tests and cigarette consumption

*Tests*	*Cigarette consumption* *(cigarettes/day)*	*Mean ± SD*	*Median (IQR)*	*p*
**Electric pulp test** (grades)	Non-smokers	4.27 ± 1.32	4 (3–5)^a^	0.001*
	1–10	5.91 ± 1.15	6 (5–7)^b^	
	11–20	6.55 ±1.1	7 (6–7)^bc^	
	20	7.23 ± 1.8	7 (6–8)^c^	
**Cold test response time** (seconds)	Non-smokers	0.51 ± 0.31	0.38 (0.3–0.66)^a^	0.001*
	1–10	0.76 ± 0.25	0.72 (0.55–0.93)^b^	
	11–20	0.94 ± 0.24	0.94 (0.72–1.05)^bc^	
	20	1.07 ± 0.33	1.02 (0.83–1.26)^c^	

* p<0.05; Kruskal Wallis test.

a,b,c : different letters represent the difference between groups. IQR: interquartile range.

Analysis of EPT responses demonstrated a clear dose-dependent increase in stimulus threshold with increasing daily cigarette consumption. Non-smokers exhibited the lowest mean EPT response values (4.27 ± 1.32), whereas light smokers (1–10 cigarettes/day) showed significantly higher values (5.91 ± 1.15). This increasing trend continued in moderate smokers (11–20 cigarettes/day: 6.55 ± 1.10) and heavy smokers (>20 cigarettes/day: 7.23 ± 1.80), with an overall statistically significant difference between groups (p=0.001). Pairwise comparisons revealed that heavy smokers had significantly higher EPT thresholds compared with non-smokers (p<0.001), indicating reduced pulpal sensibility.

Cold test results showed a similar pattern. Mean response time was shortest in non-smokers (0.51 ± 0.31 s) and increased progressively with cigarette consumption: 0.76 ± 0.25 s in light smokers, 0.94 ± 0.24 s in moderate smokers, and 1.07 ± 0.33 s in heavy smokers. These differences were statistically significant across groups (p=0.001). The median cold response time in heavy smokers (1.02 s) was nearly three times longer than that observed in non-smokers (0.38 s), highlighting a clinically relevant delay in pulpal response.

**Figure 1:**
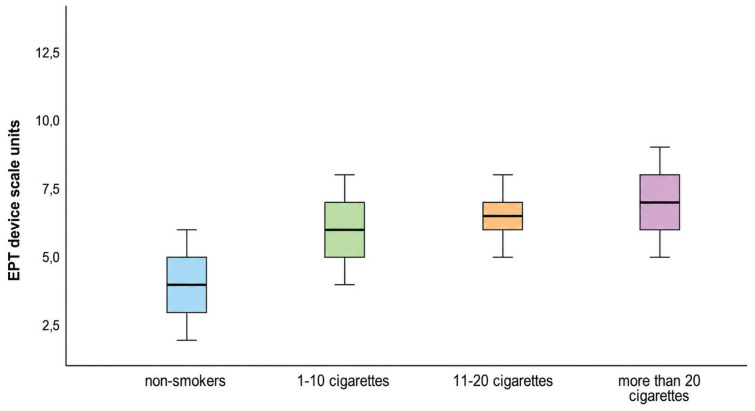
Comparison of EPT thresholds (device scale units) among smoking categories. Overall differences were assessed using the Kruskal–Wallis test, followed by Dunn’s post hoc comparisons with Bonferroni correction (p=0.001). Bars sharing the same letter are not significantly different (p>0.05)

**Figure 2:**
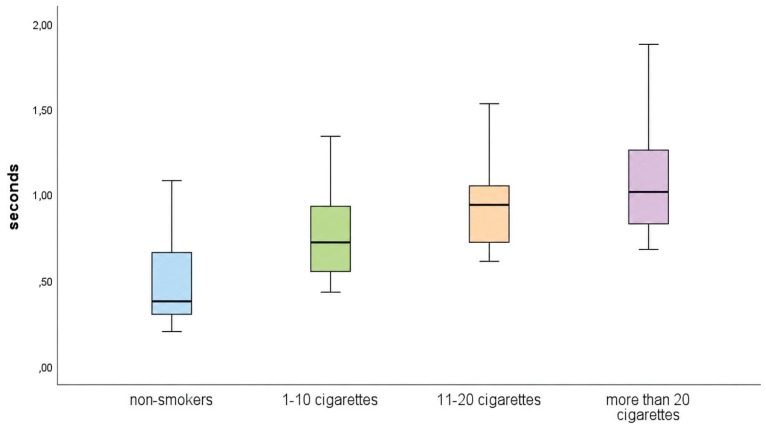
Mean cold test responses in relation to daily cigarette consumption (seconds). Overall differences were assessed using the Kruskal–Wallis test, followed by Dunn’s post hoc comparisons with Bonferroni correction (p=0.001). Bars sharing the same letter are not significantly different (p>0.05).

Figures 1 and 2 visually illustrate these findings, demonstrating a consistent upward trend in both EPT stimulus levels and cold test response times as daily cigarette consumption increased. The overall comparison revealed significant differences among smoking categories for both EPT thresholds and cold test response times (Kruskal–Wallis test, p<0.001). *Post hoc* analyses demonstrated significant pairwise differences, particularly between non-smokers and heavy smokers (>20 cigarettes/day).

Table 1 revealed no statistically significant association between the duration of the smoking habit and either EPT response grades (p=0.832) or cold test response times (p=0.258). Additionally, there was no significant relationship between smoking duration and daily cigarette consumption (p=0.315), indicating that the observed alterations in pulp sensibility were primarily related to the quantity of cigarettes consumed per day rather than the cumulative duration of smoking (Table 3).

**Table 3:** Distribution of smoking habit duration according to daily cigarette consumption

*Duration of* *smoking habit* *(years)*	*Cigarette consumption* *(cigarettes/day)*			*Total*	*p*
	*1–10*	*11–20*	*>20*		
0–5	12	9	14	35	0.315
6–10	10	13	8	31	
Total	22	22	22	66	

## DISCUSSION

Choosing the proper endodontic therapy requires evaluating the state of the dental pulp. In this study, both the cold test and EPT were employed to evaluate pulpal responses, as they are widely accepted and accessible methods in clinical endodontics. The diagnostic accuracy of these sensibility tests has been a subject of extensive research. Cold testing causes rapid outward fluid movement within dentinal tubules, stimulating A-delta nerve fibers that produce a sharp and transient pain response. In contrast, EPT generates small electrical impulses that depolarize intact myelinated nerves, triggering a response only when neural components are preserved. Therefore, EPT may yield false-negative results in cases of calcified or partially necrotic pulps, where innervation is disrupted despite the presence of vascularity^5^. In line with these findings, Farughi et al.^17^ demonstrated that sensibility tests, although indirect, offer clinically acceptable diagnostic performance when compared with vitality-based methods. Their study highlighted that the cold test exhibited the highest accuracy among sensibility assessments, while EPT provided supplementary diagnostic value. Consequently, the combined application of cold and EPT in this study was intended to increase diagnostic confidence and minimize potential misclassification of pulpal status, especially in populations with altered sensory thresholds, such as smokers^17^.

Gender-related differences in pain perception and sensory thresholds have been previously reported in the literature, potentially mediated by hormonal, neurophysiological, and psychosocial factors. For example, a systematic review indicated that females often exhibit greater sensibility to experimentally induced pain and lower pain thresholds compared with males^18^. Biological mechanisms, including hormonal influences and neural processing differences, may contribute to sex-based disparities in pain sensitivity^19^. In the present study, which was not specifically powered to detect sex-based differences in pulpal sensibility outcomes, no significant differences in EPT or cold test outcomes were observed between male and female participants.

Because of their lower EPT threshold, simpler separation, fewer caries, point connections with neighboring teeth, and higher accessibility, central maxillary incisors were chosen for sensibility testing. However, restricting the study sample to a single tooth type may limit the generalizability of the findings to other tooth groups. Anatomical and physiological differences across teeth – such as variations in root canal morphology, dentin thickness, pulp volume, vascular supply, and neural innervation – can significantly affect responses to both EPT and cold testing^5,20^. For instance, posterior teeth have more complex root structures and a higher incidence of dentinal sclerosis or secondary dentin formation due to occlusal stresses or ageing^21^. Tubular obstruction due to dentinal sclerosis or reparative dentin formation reduces dentin permeability, thus weakening the sensory response and causing conduction delay, especially in sensibility tests^1^. These factors can attenuate the sensory response or delay conduction times during sensibility tests.

While EPT does not directly assess vascular vitality, when used in conjunction with thermal tests – especially the cold test – it enhances diagnostic confidence in pulpal evaluation and supports endodontic clinical decision-making^3^. Pulpal sensibility test results, however, might be influenced by various circumstances. For instance, calcifications and nerve fiber degradation driven by age may result in delayed reactions^22^. Participants aged >40 years have been eliminated from the study to maintain confidence in the results. In addition, several health issues, such as type 2 diabetes, hypertension, anxiety, or depression^23,24^ or the use of medications such as acetaminophen and ibuprofen, may affect the response of teeth to pulp sensibility tests^25^. Those with such health conditions were also excluded from the study to ensure the reliability of the results.

Approximately 20.9% of the world population aged ≥15 years (women 7.4%, men 34.4%) used any tobacco product in 2022^6^. Smoking negatively alters the physiology of the body^26^. Tobacco smoke releases a variety of chemical contaminants, including hydrogen cyanide, carbon monoxide, and nicotine, when it comes into direct contact with oral tissues^27^. Through their stimulation of the sympathetic ganglia, adrenal medulla, and sympathetic nerve terminals, those substances increase the release of catecholamines, triggering pulp vasospasms that impair blood flow and may result in tissue impairment^28^. Under these ischemic conditions, early signs of inflammation appear in the pulp^29^. Additionally, vasoconstriction produced by smoking may lower pulpal osmotic pressure^10^. Sensory nerves of different diameters are impacted by changes in intra-pulpal pressure; pressure rises selectively block greater diameter A-delta fibers and activate smaller diameter C-fibers. A reduction in pulpal osmotic pressure may potentially have an impact on pulpal sensibility because osmotic pressure interacts with vascular permeability and blood flow in this mechanism.

In the present study, smoking status was determined solely based on selfreported cigarette consumption, specifically, whether participants had smoked at least 100 cigarettes in their lifetime or currently smoked. While this method aligns with common epidemiological definitions, it does not capture critical dimensions of smoking behavior such as duration of the habit, intensity (cigarettes per day), pattern of inhalation, or level of nicotine dependence^14,30^.

These factors have been shown to influence physiological responses and could modify pulpal reactivity to diagnostic testing. To improve classification validity, future studies should consider incorporating validated instruments such as the Fagerström test for nicotine dependence – a reliable self-report tool widely used in clinical and research settings. Additionally, measuring cotinine levels – a stable nicotine metabolite detectable in saliva, serum, or urine – provides an objective biomarker for both active and passive smoking exposure. Incorporating these measures into future protocols would enhance the precision and reproducibility of smoking classification, reduce misclassification bias, and improve comparability across studies. As a result, the internal validity of associating smoking exposure with pulpal test responses would be strengthened, providing more robust evidence for clinical and physiological interpretations.

The classification of smoking status in this study was based on internationally recognized definitions. According to the World Health Organization, a current tobacco user is defined as an individual aged ≥15 years who uses any tobacco product either daily or occasionally at the time of the survey, including cigarettes, cigars, waterpipes, and heated tobacco products^14^. Similarly, the United States Centers for Disease Control and Prevention defines a current smoker as someone who has smoked at least 100 cigarettes in their lifetime and currently smokes every day or on some days^30^. In this study, participants who reported smoking regularly or occasionally at the time of data collection were classified as current smokers, in alignment with these definitions.

Tobacco consumption can aggravate the inflammatory characteristics and promote pro-inflammatory macrophage polarization^31^. Calcitonin gene-releasing peptide, a neuropeptide, promotes pulpal inflammation and is more prevalent in the pulp of smokers than non-smokers^32^. All of those alterations may disrupt regular physiological functions, leading to disruptions in the pain pathways and various inflammatory reactions^10^. For instance, smokers and non-smokers have different taste and olfactory capacities^33^. Furthermore, people who smoke have a considerable reduction in the size of the olfactory bulb^34^. Smoking causes long-term detrimental consequences on olfactory function, including alterations in the olfactory epithelial cells and increased mortality in neurons responsible for smell. The progressive increase in EPT stimulus thresholds and cold test response times across smoking categories suggests a dose-dependent suppression of pulpal neural responsiveness. These findings indicate that the quantity of cigarettes consumed daily is a more critical determinant of altered pulp sensibility than the cumulative duration of smoking. Considering the results of our study, it is possible that similar changes in the neuronal processes related to taste and odor caused by cigarette smoke may have occurred in the pulp in a way that prolonged the response of the neural structures to sensory tests. Similar degenerative alterations in the autonomic nervous system may impair the proper function of pulp tissue^32^. The central nervous system responds to nicotine receptors in the same way as peripheral sensory organs do. As a result, while physically stimulated, smokers are less emotionally sensitive^35^. Smoking promotes pain tolerance and decreases the perception of experimental pain stimuli^36^. According to Bhandari et al.^15^, cigarette consumption and decreased pain perception are statistically correlated. All of these alterations in pain perception demonstrate the direct pain-suppressing impact of nicotine^37^. As reported by Nesbitt et al.^35^, nicotine has a direct impact on the brain processes that transmit pain. The study of Lane et al.^38^ employing electrical or thermal stimulation as well as cold pressor tests has shown that nicotine generates analgesia. The methods and findings of this study were comparable to ours, which used both cold and EPT. The capacity of nicotine to function as an agonist at neuronal nicotinic acetylcholine receptors (nAChR) is thought to be responsible for at least some of its pain-reducing properties^39^. When combined, these research results imply that nicotine produces analgesia by acting on the central nervous system.

From a different angle, our study’s findings suggest that smoking increases the time required to generate a pulp sensibility response. The reduced pain sensibility shown in the smoking condition may be due to a combination of factors, including carbon monoxide, manipulative activity, or participants’ expectations about the effects of smoking, in addition to the chemicals in tobacco^38^. Smoking is associated with chronic pain; many patients use it as a coping mechanism, and it also psychologically affects their mood.

### Limitations

This study has several limitations that should be acknowledged. Although stringent inclusion and exclusion criteria were applied to minimize confounding, residual confounding from unmeasured factors such as individual pain perception, nicotine metabolism, or subclinical pulpal alterations cannot be fully excluded. In addition, while the exclusion of passive smokers and individuals with prior dental trauma, restorations, or orthodontic treatment enhanced internal validity and ensured a homogeneous sample, it may have limited the generalizability of the findings to broader clinical populations in which such conditions are common. The study population was further restricted to systemically healthy adults aged 18–40 years, which may limit applicability to older individuals or those with systemic conditions. Due to the cross-sectional design, the observed associations between cigarette consumption and pulpal responses represent non-causal relationships and should not be interpreted as evidence of causality.

Moreover, smoking status and cigarette consumption were based on self-reported data, which may be subject to recall bias or social desirability bias. Pulpal reactions to EPT and cold stimuli were also recorded based on participant responses, which may introduce subjective variability. Another limitation relates to tooth selection. As the analysis was limited to maxillary central incisors, the findings may not be fully generalizable to posterior teeth or mandibular counterparts, given known anatomical, neural, and vascular differences. Therefore, results obtained from anterior teeth may not be directly extrapolated to premolars or molars. Future studies incorporating multiple tooth types are warranted to clarify the influence of anatomical variability on sensibility testing outcomes. With respect to statistical considerations, although an *a priori* power analysis indicated that the study was adequately powered to detect moderate-to-large effect sizes, smaller differences in pulp sensibility responses may have gone undetected. This may partly explain the absence of a significant association between smoking duration and sensibility test outcomes. Finally, although regression-based models allow adjustment for multiple covariates, confounding in the present study was primarily addressed through design-based strategies, including strict eligibility criteria and standardized testing conditions. Multivariable regression analyses and longitudinal designs in future studies could further validate and extend these findings in more diverse populations.

### Future research

This study is crucial because it serves as a basis for future research that will concentrate on the variables influencing pulpal sensibility. Clinicians should be aware that responses to pulp sensibility tests may be altered in smokers, potentially due to altered neural transmission or vascular changes. These factors can compromise the reliability of sensibility testing and lead to false-negative results even in vital pulps. Sensibility testing, especially if test results are equivocal, may be beneficial in conjunction with clinical examination, radiographic evaluation, and possibly advanced pulp vitality testing (e.g. pulse oximetry, laser Doppler flowmetry). Because thresholds may be elevated, clinicians should interpret lower-than-normal EPT values cautiously and consider repeat testing or additional diagnostic methods before initiating invasive treatment. In smokers, baseline sensibility testing at multiple time points, rather than relying on a single measurement, can help identify trend changes.

## CONCLUSIONS

Within the limitations of this cross-sectional study, daily cigarette consumption was found to be associated with altered dental pulp sensibility responses, as reflected by increased EPT thresholds and prolonged cold-test response times. In contrast, smoking duration did not demonstrate a significant association with pulpal sensibility outcomes. These findings suggest that the quantity of cigarettes consumed per day may be a more relevant indicator of modified pulpal neural responsiveness than cumulative smoking exposure. Clinically, smoking status should be considered when interpreting pulp sensibility results, as attenuated responses in smokers may increase the risk of false-negative findings. Subsequent longitudinal studies utilizing objective assessments of smoking exposure and vitality-oriented diagnostic techniques are necessary to elucidate the correlation between smoking and pulpal function.

## Data Availability

The data supporting this research are available from the authors on reasonable request.
